# Comparison of Physical Function among Elderly Japanese Women with and without Low Bone Mass and Low Muscle Mass: A Cross-Sectional Study of Older Women Who Engage in Regular Physical Activity

**DOI:** 10.3390/geriatrics7050098

**Published:** 2022-09-14

**Authors:** Tsuyoshi Katsurasako, Shin Murata, Akio Goda, Hideki Nakano, Kayoko Shiraiwa, Jun Horie, Koji Nonaka

**Affiliations:** 1 Graduate School of Health Sciences, Kyoto Tachibana University, Kyoto 607-8175, Japan; 2 Department of Rehabilitation, Koka City Minakuchi Medical Care Center, Koka 528-0049, Japan; 3 Department of Physical Therapy, Faculty of Health Sciences, Kyoto Tachibana University, Kyoto 607-8175, Japan; 4 Department of Rehabilitation, Faculty of Health Sciences, Naragakuen University, Nara 631-8524, Japan

**Keywords:** elderly women, regularly exercise, low bone mass, low muscle mass, physical function

## Abstract

A decline in physical function is common among elderly people who have lost both bone and muscle mass. The aim of this study was to investigate the relationship between low bone and muscle mass and physical function in elderly women of different age groups who exercise regularly. The analysis included 299 elderly women. Low bone mass was determined by a T-score of −2.5 or less, and low muscle mass was determined by a skeletal muscle mass index of <5.7 kg/m^2^. Physical function was measured by grip strength, knee extension strength, standing ability, gait function, and balance function. The participants were divided into four groups based on bone and muscle mass (healthy, low bone mass, low muscle mass, and low bone and muscle mass groups), and their physical functions were compared. There were no statistically significant differences in physical function between the low bone and muscle mass and the healthy groups. There were also no statistically significant differences in physical function among the four groups in the late elderly stage (75 and older). Elderly women who exercise regularly are less likely to experience a decline in physical function, even if they have reduced bone and muscle mass.

## 1. Introduction

Falls and fractures among the elderly population are strongly associated with gait disturbance, hospitalization, and mortality [1,2]; therefore, it is important to prevent their occurrence. One of the risk factors for fracture is the decrease in bone mass [3]. Bone and muscle mass decrease with aging [4], and elderly people with low bone mass often have low muscle mass [5]. Sarcopenia, which is mainly characterized by decreased muscle mass, is a risk factor for functional disability and falls [6], and a study reported that 57.3% of patients with sarcopenia had osteoporosis [7].

The coexistence of muscle and bone mass loss is described as osteosarcopenia [8], and the risk of falls and fractures is higher among the elderly population who have both than among the elderly population with bone loss or sarcopenia alone [9,10]. In addition, there is a marked interaction between bone loss and muscle loss [8], and their structure and function deteriorate with aging [11]. It is therefore important to consider both bone and muscle mass loss simultaneously to prevent disability.

Previous studies on the relationship between bone and muscle mass loss and physical function have reported that low muscle mass is associated with reduced motor function [12,13]. Decreased bone mass is reportedly associated with decreased muscle mass and strength [14], walking speed, and balance on a single leg [15]. Elderly people with decreased bone and muscle mass have decreased walking ability [16], and obese elderly women have decreased balance and walking ability [17].

Thus, bone mass and muscle mass decrease with aging, and elderly people with both losses of bone and muscle mass are likely to experience a decline in physical function. However, previous studies on physical function in older adults with both bone loss and muscle loss have focused on community-dwelling and obese older adults and have not reported on older adults who exercise regularly. Regular exercise is recommended to maintain health among the elderly population [18], and it has been reported that older adults who engage in regular exercise maintain their physical function [19]. However, the question remains as to whether the physical function can be maintained among older adults who engage in regular exercise, even if both bone mass and muscle mass have reduced. In addition, osteoporosis is significantly more common in postmenopausal older women [4], and female sex is one of the identified risk factors associated with osteosarcopenia [20]. Therefore, studies focusing on older women and identifying physical function characteristics of older women who exercise regularly and have both bone and muscle loss are important because they may provide useful information for the prevention of disability among the elderly population. In addition, since bone and muscle mass decrease with aging [11], the decline in bone and muscle mass may affect the physical function of the elderly population in the late elderly stage compared with those in the early elderly stage. Thus, it is important to investigate the relationship between bone and muscle mass decline and physical function according to the early and late elderly stages.

The purpose of this study was to investigate physical function characteristics according to the presence of low bone and muscle mass in elderly women who exercise regularly. In addition, the participants were categorized according to the early and late elderly stages to compare the differences between these groups in terms of the relationship between physical function and the occurrence of low bone and muscle mass.

## 2. Materials and Methods

### 2.1. Participants

Participants were elderly individuals who regularly performed “Iki Iki Hyakusai Taiso” [21] at least once a week and participated in physical fitness sessions held in 2018 and 2019. The total number of participants was 406. The “Iki Iki Hyakusai Taiso” [21] is a community activity in which elderly people take the initiative of maintaining their health and gather at community centers and other places to perform exercises in groups. It consists of low-intensity resistance exercises, which are performed once or twice weekly for approximately 40 min per session. In this study, participation in the exercises was confirmed by a public health nurse, and the regular participants were included for assessment.

**Figure 1:**
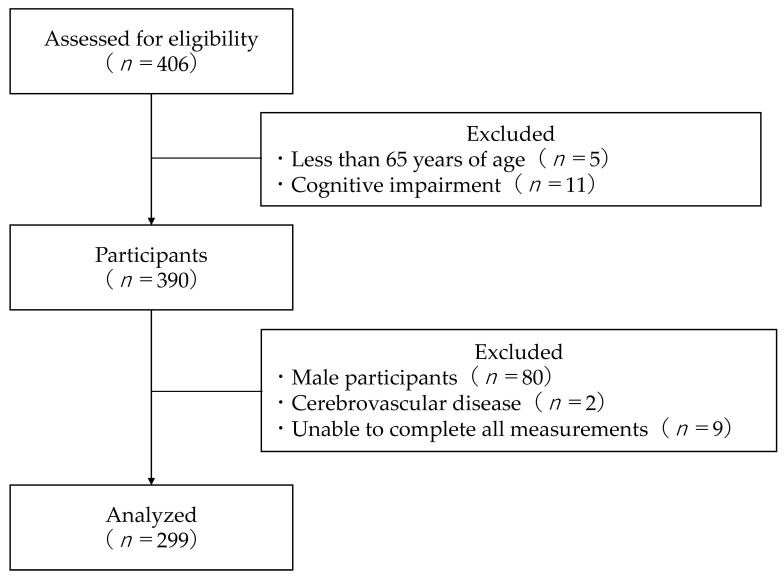
Flowchart of participation criteria.

After recording the participants’ age, height, and weight, the Mini-Mental State Examination (MMSE), an assessment of cognitive function, was administered. The inclusion criteria for this study were (1) age ≥ 65 years and (2) no cognitive impairment (MMSE ≥ 24). The exclusion criteria were (1) male sex, (2) those with a history of cerebrovascular disease, and (3) those unable to complete all measurements. After eliminating those who met the exclusion criteria, 299 of the participants were included in the analysis (Figure 1).

This study was approved by the Research Ethics Committee of Kyoto Tachibana University, Japan (Approval No. 18-26). The purpose and objectives of the study were explained to the participants in advance, and informed consent was obtained from all subjects involved in the study. Ethical considerations based on the Declaration of Helsinki were fully considered.

### 2.2. Bone and Muscle Mass Measurements

Bone mass was measured using a quantitative ultrasound device, Benus evo (Nihon Kohden Co., Tokyo, Japan), with the right calcaneus as the measurement site [22]. The World Health Organization’s T-score criteria were used for bone mass assessment; the T-score is the standard deviation compared with the average of young adults, and a T-score of −2.5 or less is considered a diagnostic criterion for osteoporosis [23]. Therefore, in this study, a T-score of −2.5 or less was defined as low bone mass and was used as a criterion for grouping the participants.

Muscle mass was measured using InBody470 (InBody Japan Inc., Tokyo, Japan), a portable body composition analyzer based on the bioelectrical impedance analysis [24]. The skeletal muscle mass index (SMI) was calculated by dividing the skeletal muscle mass of the limb obtained from the measurements by a square of the participant’s height. An SMI of <5.7 kg/m^2^ in women was defined as low muscle mass, following the criteria of the Asian Sarcopenia Working Group (2019) [25].

### 2.3. Measurement of Physical Function

Physical functions were measured by grip strength, knee extension strength, time to stand on one leg with eyes open, 5 m fastest walk, timed up-and-go test (TUG), and 30 s chair rise test (CS-30). The grip strength was measured using a digital grip strength meter (T.K. K.58401, Takei Scientific Instruments Co., Ltd., Niigata, Japan), and the grip strength was adjusted such that the second joint of the index finger was at the right angle. The participants were instructed to stand with their legs naturally spread to the left and right and their arms hanging down and to grip with maximum force while preventing the grip strength meter from touching their bodies [26]. Measurements on each side were performed twice, and the maximum value was divided by the body weight.

Knee extension strength was measured using a hand-held dynamometer (μ- Tas F-1; Anima Corp., Tokyo, Japan) in a chair-sitting position with a belt attached to the chair post and an isometric muscle strength in the lower leg drop position. Measurements were taken with the participants in a chair-sitting posture with the hip and knee joints in 90° flexion, and the participants were instructed to extend the knee joint with maximal effort [27]. Measurements on each side were performed twice, and the maximum value divided by body mass was used.

“Time to stand on one leg with eyes open” measurement was performed using the method described by MacRae et al., with slight modifications [28]. The duration between the time one foot is raised to the time that foot touches the ground was measured with a digital stopwatch with the participants barefoot and with both hands to their sides. The maximum measurement time was set to 120 s. Measurements were performed twice on each side, and the longest time was used as the measured value.

The 5 m fastest walking time was measured by setting up a 5 m walking path (at the measurement section) and a 3 m auxiliary path at both ends, and the 5 m walking time was measured with a digital stopwatch. The participants were instructed to walk with maximum effort as fast as possible [29]. Measurements were performed twice, and speed was calculated by dividing 5 m by the fastest walking time.

The TUG was measured using a chair with a backrest. A digital stopwatch was used to measure the time taken to stand from a sitting posture with the back against the backrest, walk forward as fast as possible with maximum effort, turn around a landmark placed 3 m in front of the chair, and sit down on the chair again [30]. The measurements were performed twice, and the shortest time was used as the measured value.

The CS-30 was measured using a chair with a height of 40 cm, and the starting limb position was a sitting posture with arms crossed in front of the chest. The participants were instructed to stand from this posture as fast as possible and then sit down again. The number of times the participant was able to stand in 30 s was recorded [31].

### 2.4. Statistical Analysis

The participants in this study were classified into the following four groups: healthy group (T-score > −2.5, SMI ≥ 5.7 kg/m^2^), low bone mass group (T-score ≤ −2.5, SMI ≥ 5.7 kg/m^2^), low muscle mass group (T-score > −2.5, SMI < 5.7 kg/m^2^), and low bone and muscle mass group (T-score ≤ −2.5, SMI < 5.7 kg/m^2^). Age, height, weight, grip strength, knee extension strength, gait speed, TUG time duration, CS-30 score, and open-eyed one-leg standing time were compared among these four groups. First, a Shapiro–Wilk test was performed to confirm the normality of the data. One-way analysis of variance (ANOVA) was performed for normally distributed data, and multiple comparisons using the Bonferroni correction test were subsequently performed for variables that showed statistically significant differences among the four groups when one-way ANOVA was performed. For non-normally distributed data, the Kruskal–Wallis test was performed, and multiple comparison tests using Dunn’s test were performed. To assess physical function, the effect size, r, was calculated between the healthy group and the other groups. The strength of the effect size was considered small (<0.30), moderate (0.30–0.49), or large (>0.5) [32]. Furthermore, the participants were divided into two age groups, early elderly (65–74 years), and late elderly (75 years and older) groups, and these were compared. Statistical analysis was performed using SPSS version 28.0 (IBM Japan, Ltd., Tokyo, Japan) at a 5% significance level.

## 3. Results

The basic attributes and physical function characteristics of the participants according to the four groups in this study are shown in Table 1. There were 143 (47.8%) participants in the healthy group, 59 (19.7%) in the low bone mass group, 56 (18.7%) in the low muscle mass group, and 41 (13.7%) in the low bone and muscle mass group. The variables that showed significant differences among the four groups were height (*p* = 0.007) and weight (*p* < 0.001); there were no significant differences among the four groups in grip strength, knee extension strength, walking speed, TUG, CS-30, and open-eyed one-leg standing time.

Table 2 and Table 3 show the physical function comparison between participants in the early and late elderly stages. For each physical function variable, there were no statistically significant differences among the four groups in both the early and late elderly groups.

The effect size obtained by comparing the physical function of the healthy group with the other groups is shown in Table 4 and Table 5. The overall effect size of the differences between the healthy group and the other groups as well as according to the early and late elderly stages were small.

## 4. Discussion

This study investigated physical function characteristics according to the presence or absence of low bone and muscle mass in elderly women who exercise regularly, which has not been reported in previous studies. There were no significant differences in physical function among the four groups in this study. In addition, there was no significant difference in physical function between the healthy group and the other groups. Furthermore, all the effect sizes of the differences between the healthy and the other groups were small. The results suggest that, although previous studies on community-dwelling older adults and obese older women have reported that older adults who have lost both bone mass and muscle mass have reduced physical function, older women who exercise regularly maintain their physical function even when both bone mass and muscle mass are reduced.

In previous studies of community-dwelling elderly and elderly obese females, those with both low bone and muscle mass had poorer physical function [16,17]. The results of the present study differed from those of the abovementioned previous studies. A characteristic of this study that differentiates it from previous studies is that it included elderly people who regularly engaged in exercise. Strasser et al. reported that the elderly participants who performed resistance exercises twice a week for 6 months showed improvement in muscle strength but no change in muscle mass [33]. It has also been noted that muscle strength is not only related to muscle mass but also to muscle quality [34]. The results of this study suggest that there were no significant differences in muscle strength among the four groups, presumably because muscle quality was preserved through regular exercise even though muscle mass was reduced with aging. Previous studies on physical function and muscle strength have reported that lower limb muscle strength is associated with the walking ability [24,35], CS-30 [36], and open-eyed one-legged standing time [37] among the elderly population. In this study, knee extension strength, which was used as an index of lower limb muscle strength, did not differ significantly among the four groups, suggesting that there were no significant differences in walking ability, CS-30, or open-eyed one-legged standing time. The results of this study suggest that elderly people who engage in regular exercise can maintain their physical functions even when both bone and muscle mass have declined.

We divided the participants into early and late elderly stages and compared the physical function of the elderly population with and without low bone and muscle mass. However, among the four groups, no significant differences were found in each physical function variable for participants in the early or late elderly stages, and all the effect sizes were small. Bone loss and muscle loss interact, and their structure and function decline with aging [11]. The prevalence of osteosarcopenia in elderly people living in the community increases with aging and is reported to be more strongly associated with women than with men [8,20]. Therefore, it can be inferred that late-elderly-stage female patients with both bone and muscle mass loss are more likely to experience a decline in physical function. However, when the present study focused only on the late elderly stage, physical function was maintained even with decreased bone mass and muscle mass.

This study suggests that, among the elderly population who engage in regular exercise, the group with reduced bone and muscle mass can maintain the same level of physical function as that observed in the healthy group, even with increasing age.

In this study, the participants were highly active elderly people who participated in community activities. In the community projects where the participants reside, activities are centered on the “Iki Iki Hyakusai Taiso” [21]. “Iki Iki Hyakusai Taiso” is held once or twice a week at a community center and consists mainly of low-intensity upper and lower limb strengthening exercises lasting for approximately 40 min [21]. Although high loads are considered ideal for resistance exercises that aim to increase muscle strength, it has been shown that even low loads can be effective in increasing the muscle strength of the elderly population [38]. The effects of resistance exercise are not permanent, and improvements such as improvement in muscle strength disappear after a short period of exercise [39,40]. In this study, we inferred that regular and continuous exercise, even at low loads, maintained physical function in the group with reduced bone and muscle mass.

The elderly patients in this study with both low bone and muscle mass showed a significantly lower body weight but retained physical function such as walking ability and balance, similar to that in the healthy group. Physical function was also maintained even in the elderly population in the late elderly stage, who are more susceptible to the effects of aging. The results suggest that elderly people with both bone and muscle mass loss are likely to experience physical function decline but that they can maintain their physical function by continually performing exercise, even at low loads. These findings can provide important information for efficient physical activity promotion strategies targeted at health promotion and disability prevention among the elderly population.

This study has some limitations. First, it is a cross-sectional study; therefore, causal relationships cannot be established. Second, although the participants were elderly women who exercised regularly, we were unable to conduct a detailed study of the intensity and duration of the exercise performed, nor assess the duration of their participation. Future, longitudinal studies including elderly women who do not exercise regularly, should be conducted to examine the effects of the differences in exercise intensity, duration of exercise, and duration of participation on physical function in older adults who have lost both bone mass and muscle mass.

## 5. Conclusions

In this study, we investigated the relationship between the physical function of elderly Japanese women who exercise regularly and the presence of bone and muscle mass loss. No significant differences in physical function were found between the groups with muscle mass loss, bone mass loss, or bone and muscle mass loss and the healthy group. This study suggests that elderly women who exercise regularly are less likely to experience a decline in physical function, even if they have decreased bone mass and muscle mass.

## Figures and Tables

**Table 1:** Comparison of basic attributes and physical function among the four groups.

	A, Normal (*n* = 143)	B, Low Bone Mass (*n* = 59)	C, Low Muscle Mass (*n* = 56)	D, Low Bone and Muscle Mass (*n* = 41)	*p*	Multiple Comparison
Age (years)	73.6 ± 5.3	73.9 ± 5.1	74.6 ± 5.9	75.2 ± 5.7	0.464	
Height (cm)	152.0 ± 5.5	151.3 ± 5.0	149.5 ± 4.8	149.6 ± 5.8	0.002	A < C
Weight (kg)	53.1 ± 6.5	52.5 ± 6.3	44.7 ± 5.0	45.1 ± 4.3	<0.001	AB > CD
Grip strength (kgf/kg, %)	47.2 ± 8.3	47.3 ± 8.0	48.2 ± 9.7	48.2 ± 7.8	0.838	
Knee extension strength (kgf/kg, %)	39.5 ± 8.9	40.8 ± 10.4	40.7 ± 7.6	41.3 ± 9.9	0.405	
Fastest gait speed (m/s)	1.88 ± 0.26	1.83 ± 0.27	1.77 ± 0.29	1.81 ± 0.25	0.085	
TUG (s)	5.7 ± 0.9	5.7 ± 1.2	5.9 ± 1.4	5.8 ± 0.9	0.752	
CS-30 (times)	21.4 ± 5.7	21.8 ± 6.4	21.5 ± 5.8	20.7 ± 4.7	0.785	
One-leg standing (s)	39.5 ± 35.8	42.3 ± 34.0	37.1 ± 41.7	33.8 ± 36.7	0.099	

Values are presented as means ± SD. TUG, Timed up and go test; CS-30, 30-s chair stand test.

**Table 2:** Comparison of physical functions of elderly participants in early elderly stage among the four groups.

	A, Normal (*n* = 86)	B, Low Bone Mass (*n* = 35)	C, Low Muscle Mass (*n* = 31)	D, Low Bone and Muscle Mass (*n* = 21)	*p*	Multiple Comparison
Grip strength (kgf/kg, %)	47.9 ± 7.8	47.1 ± 6.5	49.1 ± 9.0	48.4 ± 8.1	0.782	
Knee extension strength (kgf/kg, %)	40.0 ± 8.3	42.2 ± 1.0	41.5 ± 5.3	41.7 ± 7.8	0.37	
Fastest gait speed (m/s)	1.94 ± 0.24	1.92 ± 0.23	1.86 ± 0.26	1.85 ± 0.21	0.284	
TUG (s)	5.5 ± 0.7	5.3 ± 0.7	5.3 ± 0.8	5.6 ± 0.6	0.487	
CS-30 (times)	22.2 ± 5.4	22.8 ± 6.0	22.5 ± 6.3	20.6 ± 3.7	0.542	
One-leg standing (s)	49.9 ± 38.8	54.0 ± 35.3	46.9 ± 43.0	39.7 ± 39.7	0.18	

Values are presented as means ± SD. TUG, Timed up and go test; CS-30, 30-s chair stand test.

**Table 3:** Comparison of physical functions of elderly participants in late elderly stage among the four groups.

	A, Normal (*n* = 57)	B, Low Bone Mass (*n* = 24)	C, Low Muscle Mass (*n* = 25)	D, Low Bone and Muscle Mass (*n* = 20)	*p*	Multiple Comparison
Grip strength (kgf/kg, %)	46.1 ± 9.0	47.7 ± 9.9	47.0 ± 10.6	48.0 ± 7.6	0.838	
Knee extension strength (kgf/kg, %)	38.6 ± 9.7	38.9 ± 10.3	39.6 ± 9.8	40.9 ± 11.9	0.833	
Fastest gait speed (m/s)	1.78 ± 0.26	1.70 ± 0.28	1.66 ± 0.28	1.77 ± 0.29	0.271	
TUG (s)	6.0 ± 1.1	6.4 ± 1.5	6.7 ± 1.6	6.0 ± 1.2	0.242	
CS-30 (times)	20.2 ± 6.3	20.7 ± 5.6	20.1 ± 4.9	20.7 ± 5.6	0.985	
One-leg standing (s)	24.0 ± 23.3	25.3 ± 23.6	25.0 ± 37.3	27.5 ± 33.0	0.601	

Values are presented as means ± SD. TUG, Timed up and go test; CS-30, 30-s chair stand test.

**Table 4:** Effect sizes of the differences between the healthy group and the other groups.

	Low Bone Mass	Low Muscle Mass	Low Bone and Muscle Mass
Grip strength	0.01	0.05	0.05
Knee extension strength	0.06	0.1	0.1
Fastest gait speed	0.07	0.17	0.1
TUG	0.02	0.05	0.06
CS-30	0.01	0.03	0.08
One-leg standing	0.07	0.11	0.11

TUG, Timed up and go test; CS-30, 30-s chair stand test.

**Table 5:** Effect sizes of the differences between the healthy group and the other groups in the early and late elderly changes.

		Low Bone Mass	Low Muscle Mass	Low Bone and Muscle Mass
Early elderly	Grip strength	0.05	0.07	0.03
	Knee extension strength	0.11	0.14	0.09
	Fastest gait speed	0.04	0.14	0.15
	TUG	0.01	0.07	0.05
	CS-30	0.04	0.02	0.13
	One-leg standing	0.1	0.08	0.14
Late elderly	Grip strength	0.08	0.05	0.1
	Knee extension strength	0.03	0.06	0.09
	Fastest gait speed	0.13	0.2	0.02
	TUG	0.11	0.2	0
	CS-30	0.02	0	0.04
	One-leg standing	0.01	0.15	0.04

TUG, Timed up and go test; CS-30, 30-s chair stand test.

## Data Availability

Not applicable.
